# Supplementary material for: Double jeopardy: global change and interspecies competition threaten Siberian cranes
Source: PeerJ. 2024 Feb 28;12:e17029. doi: 10.7717/peerj.17029 (PMC10908270; doi:10.7717/peerj.17029)
Supplement: Supplemental Information 2 [file peerj-12-17029-s002.pdf]

Supplementary 2

Response curves for for Siberian crane (*Leucogeranus leucogeranus*) and sandhill crane (*Grus canadensis*).

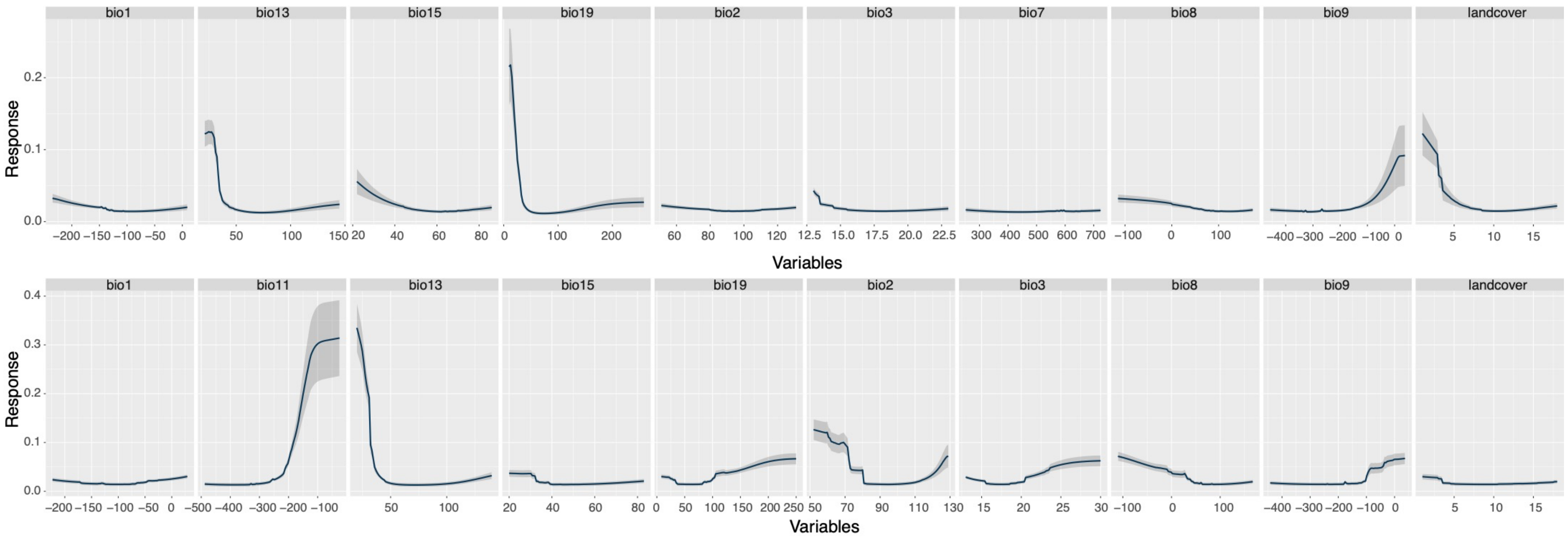

bio1 = Annual Mean Temperature, bio2 = Mean Diurnal Range, bio3 = Isothermality, bio7 = Temperature Annual Range, bio8 = Mean Temperature of Wettest Quarter, bio9 = Mean Temperature of Driest Quarter, bio11= Mean Temperature of Coldest Quarter, bio13 = Precipitation of Wettest Month, bio15 = Precipitation Seasonality, bio19 = Precipitation of Coldest Quarter, landcover = land cover.
